# Improving Carers’ Engagement in Patient Care During Psychiatric Admissions

**DOI:** 10.1192/bjo.2026.11258

**Published:** 2026-06-30

**Authors:** Nnamdi Anyim, Ashleigh Grindey, Carolyn Asher

**Affiliations:** Greater Manchester Mental Health (GMMH) NHS Foundation Trust, Manchester, United Kingdom

## Abstract

**Aims:**

Carers are important stakeholders in the recovery journey of patients admitted to inpatient psychiatry units. Often carers report not being involved in the admission journey and recovery process of those they care for. This sometimes leads to bad outcomes for patients and complaints from carers. On Keats ward, Meadowbrook Unit – an adult female inpatient acute psychiatric unit, we had multiple incidents of career dissatisfaction and complaints which birthed the idea of this quality improvement project. A baseline audit in October 2024 showed a general poor performance at key stages of carers’ engagement during the admission process. The aim of this project was to develop, test and implement a uniform tool to facilitate, capture and document carers engagement during different stages of the patient’s journey.

**Methods:**

Prior to onset of the project, the project team met repeatedly to identify thematic areas where career engagement could be improved. Ten key areas were initially identified.These ten key areas were presented to patient groups and carer representatives in focused group discussions. From these discussions, the thematic areas were reduced to five key areas from which we formed our audit tool. The project team working with senior leadership and ward staff, developed and implemented a standardised Carer Contact Form as a tool to collect, document and facilitate carer engagement during a patient’s admission. Implementation took place over several PDSA cycles between November 2024 and July 2025, supported by regular re-audits, staff training and competency assessments for nursing assistants.

**Results:**

Re-audit results after the first nine months of implementation showed marked improvements in all domains including: carer notified of admission (30% to 87%), adocumented carer care plan (50% to 60%), obtaining collateral information from carer (30% to 87%), and notifying carers of patient discharge (40% to 80%). Nursing assistant staff were trained to use the carer contact form and staff reported greater confidence in engaging with carers and there were fewer complaints from families.

**Conclusion:**

We found that the carer’s contact form was a simple and effective tool in improving carer’s engagement during the admission process on the ward. Carers’ engagement improved massively across all the indicators. Ward staff reported generally that it supported them greatly and improved their confidence in engaging with carers. A downside we worried about was creating more paperwork for staff; however, staff generally found it more helpful than problematic. We have received fewer complaints from carers and relatives about not being involved in admissions and discharges, and other wards within the unit have adopted and implemented this tool.

